# Cerebral Microcirculation and Histological Mapping After Severe Head Injury: A Contusion and Acceleration Experimental Model

**DOI:** 10.3389/fneur.2018.00277

**Published:** 2018-05-07

**Authors:** Judith Bellapart, Kylie Cuthbertson, Kimble Dunster, Sara Diab, David G. Platts, Owen Christopher Raffel, Levon Gabrielian, Adrian Barnett, Jenifer Paratz, Rob Boots, John F. Fraser

**Affiliations:** ^1^Department of Intensive Care, Royal Brisbane and Women’s Hospital, Brisbane, QLD, Australia; ^2^Department of Histopathology, Royal Brisbane and Women’s Hospital, Brisbane, QLD, Australia; ^3^Critical Care Research Group, University of Queensland, Brisbane, QLD, Australia; ^4^Medical Engineering Research Facility, Queensland University of Technology, Brisbane, QLD, Australia; ^5^Department of Cardiology, The Prince Charles Hospital, Chermside, QLD, Australia; ^6^Medical School, University of South Australia, Adelaide, SA, Australia; ^7^Medical Research Centre, Adelaide, SA, Australia; ^8^Institute of Health and Biomedical Innovation & School of Public Health and Social Work, Queensland University of Technology, Brisbane, QLD, Australia; ^9^Department of Intensive Care, The Prince Charles Hospital, Chermside, QLD, Australia

**Keywords:** anemia, amyloid precursor protein staining, histology, microcirculation, microspheres

## Abstract

**Background:**

Cerebral microcirculation after severe head injury is heterogeneous and temporally variable. Microcirculation is dependent upon the severity of injury, and it is unclear how histology relates to cerebral regional blood flow.

**Objective:**

This study assesses the changes of cerebral microcirculation blood flow over time after an experimental brain injury model in sheep and contrasts these findings with the histological analysis of the same regions with the aim of mapping cerebral flow and tissue changes after injury.

**Methods:**

Microcirculation was quantified using flow cytometry of color microspheres injected under intracardiac ultrasound to ensure systemic and homogeneous distribution. Histological analysis used amyloid precursor protein staining as a marker of axonal injury. A mapping of microcirculation and axonal staining was performed using adjacent layers of tissue from the same anatomical area, allowing flow and tissue data to be available from the same anatomical region. A mixed effect regression model assessed microcirculation during 4 h after injury, and those results were then contrasted to the amyloid staining qualitative score.

**Results:**

Microcirculation values for each subject and tissue region over time, including baseline, ranged between 20 and 80 ml/100 g/min with means that did not differ statistically from baseline flows. However, microcirculation values for each subject and tissue region were reduced from baseline, although their confidence intervals crossing the horizontal ratio of 1 indicated that such reduction was not statistically significant. Histological analysis demonstrated the presence of moderate and severe score on the amyloid staining throughout both hemispheres.

**Conclusion:**

Microcirculation at the ipsilateral and contralateral site of a contusion and the ipsilateral thalamus and medulla showed a consistent decline over time. Our data suggest that after severe head injury, microcirculation in predefined areas of the brain is reduced from baseline with amyloid staining in those areas reflecting the early establishment of axonal injury.

## Introduction

Severe head injury is commonly the result of a combination of contusion with acceleration–deceleration forces leading to cellular breakdown, cytogenic and vasogenic edema, impaired cerebral autoregulation, and perfusion mismatch (1). Irreversible cellular damage has been described specifically in areas where microcirculation is critically reduced (2, 3) and in areas where critically low levels of partial pressure of tissue oxygenation (PtiO_2_) have been sustained (4). While the assessment of cerebral regional microcirculation is primordially experimental in nature, its knowledge is essential in comprehending further the pathophysiology behind severe head injury and its extrapolation to clinical grounds. In the interim, management of head injury patients is commonly based on systemic measures that ensure global perfusion and oxygenation, without specifically targeting cerebral metabolic demands, cerebral tissue oximetry, or regional distribution of blood flow. Despite the previously demonstrated relationship between regional microcirculatory blood flow (RMBF), tissue metabolic demands (5), and cerebral hypoperfusion leading to irreversible cellular damage, there are no studies quantifying the temporal variability of RMBF in different cerebral anatomical regions after severe head injury, its close relation to the degree of tissue damage assessed by amyloid precursor protein (APP) staining or the state of cerebral tissue oxygenation. We hypothesized that after severe head injury cerebral microcirculation heterogeneity may correlate to the severity of the injury with maximal RMBF reduction seen within the areas of severe tissue disruption. We also hypothesized that RMBF and APP show a temporal and spatial distribution, after injury.

This study aims to quantify the temporal changes in cerebral RMBF after severe head injury focusing on the anatomical region such as the ischemic penumbra in comparison with contralateral regions. This study also aims to simultaneously superimpose flow data with histopathology data at each area of interest and at each time point before and after severe head injury to establish the hypothetical pathophysiological relation between perfusion and mechanical related injury to axons.

## Materials and Methods

### Animal Care and Preparation

Sheep were considered to be the optimal experimental model due to their cerebral anatomical similarities with humans, specifically the cerebral gyrencephalic surface allowing better examination of the gray–white matter; a well-defined physiology of the ovine hemoglobin dissociation curve (6) and extensive experimental neuroscience experience using this animal model (7–9). A convenience sample of eight Ovis Aries wethers weighing 40 ± 5 kg were instrumented with a triple lumen central line (Cook Medical Inc., QLD, Australia) and two 16 Fr introducer sheaths in the right internal jugular vein. *Via* the central line, general anesthesia was given using ketamine with an initial bolus of 5 mg/kg and maintenance infusion between 0.5 and 1 mg/kg/h. Sedation was achieved with a combined infusion of midazolam (0.5 mg/kg/h), fentanyl (10 μg/kg/h), and alfaxalone (6 mg/kg/h), previously used in a mild head injury study (10) demonstrating that this anesthetic combination maintains cardiovascular stability without altering cerebral microcirculation in sheep (11). Hydration was maintained with an infusion of Hartmann’s solution up to a rate of 2 mL/kg/h, titrated to maintain a central venous pressure of 6–10 mmHg. Cardiovascular monitoring included cardiac output (CO) and vascular resistances *via* a Swan-Ganz catheter as previously described (10, 12) and a 5 F umbilical vessel catheter (Argyle, Tyco HealthCare, Mansfield, MA, USA), placed in the right femoral artery to allow a withdrawal of blood at a rate of 10 ml/min. Orotracheal intubation used a size 10 mm endotracheal tube (SIMS Portex, UK). Sheep were ventilated at 12 bpm with tidal volumes of 8 mL/kg and 5 cmH_2_O of PEEP with an initial FiO_2_ of 1.0 with the FiO_2_ and respiratory rate titrated to maintain a partial pressure of oxygen (PaO_2_) of >95 mmHg and normocapnia. PEEP levels were maintained at 5 cmH_2_O to minimize de-recruitment consistent with common clinical practice and known to have no detrimental effect on cerebral blood flow (10, 13). Neuro-monitoring included a Lycox PtiO_2_ probe and an intracranial pressure (ICP) monitor, Oxford Optronix Ltd., Oxford, UK. Craniectomies were performed before injury but dura was not pierced to avoid the ICP release if pierced beforehand. Craniotomies for the insertion of both probes were performed exactly 15 mm lateral to the sagittal suture and anterior to the coronal suture (9, 14). Probes were introduced at 35 and 15 mm from the skull, respectively, after piercing the dura with the end of the tip located at the white matter as previously performed (15).

To avoid red blood cell storage into the sheep’s spleen and maintaining stable hemoglobin throughout the study (16, 17), the ligation of the splenic artery was performed as reproduced from previous studies (10, 15).

The monitoring and preparation phase was completed with an intracardiac echocardiography guided insertion of a transeptal catheter into the left cardiac atrium (LA). Echocardiography images were obtained using an Acuson Sequoia C512 scanner (Siemens, California). Transeptal puncture and insertion of a pigtail catheter into the LA followed previously described methods (18).

### Trauma Model

Under anesthesia, a blunt injury was applied over the left temporal bone using a non-penetrating stunner (model MKL, Karl Schermer, Ettlingen, Germany) with the intention to generate a severe head injury but without leading to brain death. This model followed the exact method as a previous study (10) leading to a final combination of contusion and acceleration injury (19). After injury, burr holes were formalized and the dura was pierced for the insertion of pressure and tissue-oximetry probes as previously described (10). The main difference with the previously reported study (10) was that in this study the goal was to achieve a severe head injury, unlike the former study which aimed to achieve a mild degree of head injury. To achieve this goal, recruited animals were significantly smaller in weight: while for the achievement of a “mild” head injury, animals weighed between 60 and 75 kg, in this study, recruited animals weighed between 40 and 45 kg. Subjects were still adult sheep and wethers of the same ovine species. As the impact forces generated by the same stunner were unchanged; the result of these forces onto smaller brains was expected to be different. This latter supposition is based upon the theoretical rationale that a reproducible and identical force may lead to greater impact or harm when applied to a smaller area.

### Protocol for Microspheres Injection

At each time point (T0 corresponding to baseline, T1–T4 corresponding to first to fourth hours after trauma, respectively) an injection of color-coded microspheres [E-Z TRAC; Interactive Medical Technology (IMT), Los Angeles, CA, USA] was done through the LA pigtail catheter as performed previously (10, 15, 20). Randomly assigned colors at each time point and subject minimized selection biases and allowed the tracking of RMBF at specific anatomical regions for each time point and subject. Five different colors (purple low, purple high, pink high, yellow high, and coral low) were recommended by the manufacturer www.microspheres.net to facilitate cytometric count. Each injection included a homogeneous mixture of one color microspheres of a density equal to five million spheres in a 0.8 ml. This microsphere density has been used (21) without causing microvascular occlusion.

Microspheres were injected 30 s after the initiation of the withdrawal pump. The withdrawal pump was connected to the arterial catheter with the intention to withdraw blood at an established rate of 10 ml/min to obtain the reference blood sample required for the calculation of regional tissue RMBF. Two minutes after commencement of the withdrawal pump, the reference blood sample collection was completed, and the inline catheter was flushed with Tween 80 reagent to recover all the microspheres that may have been entrapped in the line (22). This protocol reproduced the same steps as previously published studies (10, 15).

### Euthanasia and Postmortem Tissue Manipulation

After 5 h of continuous monitoring and microsphere injection, sheep were euthanized under non-recovered anesthesia with a bolus injection of 0.5 mL/kg of sodium pentobarbitone. After confirmation of death (asystole arrest), the brain was extracted, weighed, and fixed with 10% formalin for 3 weeks.

### Brain Harvesting Technique

Brain harvesting was facilitated with the use of a round reciprocating saw sectioning approximately 5 cm bone sections from the temporal region to the frontal sinuses. These bone sections were removed while simultaneous dissection of the dura avoided parenchymal tearing. Once the brain was fully exposed, the olfactory bulbs, optic chiasm, tentorium, and cranial nerves were progressively sectioned as the brain was lifted from the base of the skull. This approach achieved a controlled dissection avoiding injury to the brain tissue and ensuring a cautious brain removal as previously demonstrated (11). Brains were weighed before insertion into a formalin bath for an immersion fixation during a minimum of 3 weeks.

### Tissue Sampling Model

After the period of immersion fixation, brains were macroscopically inspected to assess for cortical impacts, hemorrhages, or the presence of contra-coup injury. Following external inspection, each brain was sectioned creating 5 mm anteroposterior slices. Each slice was macroscopically inspected to identify regions of maximal contusion. Cone samples were extracted from predefined anatomical regions these labeled as follows: AL corresponding to the core of contusion at the side of the injury; BL, the peri-contusional region at the side of the injury; AR, the mirror region to the core of contusion on the contralateral side; BR, the mirror region to the peri-contusional region on the contralateral side; C, the thalamus at the ipsilateral side to the contusion, and D corresponded to the medulla (Table 1), this method was reproduced from a previous study (10, 15). Adjacent tissue blocks were assigned for both cytometric and histological analysis, to superimpose histology with cerebral blood flow data at the same anatomical region.

**Table 1:** Tissue sampling labeling.

Anatomical regions	Anatomical location
AL	Core of contusion, left side
BL	Ischemic penumbra, left side
AR	Mirror region to core of contusion, on the right
BR	Mirror region to ischemic penumbra, on the right
C	Thalamus ipsilateral to injury
D	Medulla

Samples from skin, kidney, heart, and spleen were extracted from each sheep to demonstrate systemic distribution of microspheres as well as to confirm the presence of splenic infarct related to a successful spleen ligation, respectively.

### Quantification of Microvascular Blood Flow

The total amount of each color microsphere imbedded in each particular region of interest used a cytometric analysis which has been previously validated (22). RMBF was calculated as a mathematical derivation from the known microsphere concentration injected into the arterial supply at each injection time and the amount of each color microspheres found at each “reference sample of blood.” The reference sample of blood represents an arterial blood sample withdrawn at a known rate over a fixed period of time (23). RMBF represents the proportion of microspheres trapped in the targeted tissue in relation to the total quantity of spheres per milliliters of blood per minute of the reference sample using the equation (24):

$$\begin{array}{l} \text{RMBF }(\text{mL}/\text{min}/\text{g})=(\text{Total tissue spheres})\\ /\left[\left(\text{Tissue weight},\text{ g})\times (\text{Reference spheres}/\text{mL}/\text{min}\right)\right]. \end{array}$$

Cytometric analysis was performed at the IMT, Los Angeles, CA, USA, www.microspheres.net.

### Immunohistochemistry Processing

Immunohistochemistry analysis was performed at the neuropathology laboratory, Royal Brisbane and Women’s Hospital, QLD, Australia. Immunohistochemistry used a Leica Novolink Polymer Detection Systems Kit (Leica Microsystems Pty Ltd., North Ryde, NSW, Australia) as per manufacturer’s instructions, www.leica-microsystems.com. Sections had paraffin removed through a series of xylene immersions and re-hydrations. Antigen retrieval was carried out using Leica BOND ER1 solution. Endogenous peroxidize was neutralized. Sections were incubated with a protein block. The primary antiserum made up in Leica BOND Antibody Diluent was applied to the sections.

### Immunohistochemistry and Hematoxylin–Eosin Scoring and Interpretation

Immunohistochemistry analysis using APP antibodies staining was applied to all targeted areas of interest. APP antibody staining was used to identify areas of tissue with high density of APP staining, specifically at regions of interest. APP expression is considered to be a very early marker of neuronal damage (25) and therefore suitable as an early histopathological marker for a 4 h study. A grading system of the density of APP staining previously described (9) was used. APP score was structured into three qualitative categories dependent upon the severity of injury seen: *Mild*: a focal contusion with APP labeling limited to the site of injury or focal APP labeling; *Moderate*: a pattern of APP staining greater than one hemisphere, greater than half a hemisphere or less than half a hemisphere; *Severe*: characterized for the presence of diffuse staining and sub-classified as either diffuse vascular injury, diffuse axonal injury with macroscopic hemorrhage, diffuse axonal injury with microscopic hemorrhage, tissue tears or diffuse axonal injury only (11). Each animal had samples for both cytometric count of RMBF and immunohistochemistry at each anatomical region of interest with the intention to superimpose flow data with histopathology data at each area of interest and at each time point before and after severe head injury.

### Statistical Analysis

Regional microcirculatory blood flow raw data for each sheep was plotted over time and then time averaged for the study cohort. The ratio of RMBF from 1 to 4 h after injury (T1–T4) compared with baseline (time 0—T0) was plotted. A ratio of 1 indicated no RMBF changes pre–post-injury. RMBF values below the ratio of 1 indicted that RMBF was reduced over time from baseline. RMBF values above the ratio indicated an increase in RMBF over time from baseline. To test for statistical differences, we used a mixed effect regression model of the ratios from times T1 to T4 with a random intercept for each sheep to control for repeated responses from the same sheep. We fitted an independent effect at each time (T1–T4) as we were uncertain of how the change in RMBF over time would be. We also examined a simpler model where the RMBF ratio was the same at times T1–T4.

All the plots and regression models were run separately for each area studied (AR, BR, AL, BL, C, and D). We used the R software version 3.1.2 for all analyses.

An additional analysis was performed to compare RMBF ratios from a “mild head injury” cohort used in a previous study (10) and the current “severe head injury” cohort study. We plotted the mean ratio over time grouped by study to graphically compare differences. To test for statistical differences, we used a mixed effect regression model of the ratios from times T1 to T4 with a random intercept for each sheep to control for repeated responses from the same sheep. We fitted an independent effect at each time (T1–T4); the key independent variable was the study (the “mild head injury” cohort study versus the “severe head injury” cohort study). The mean difference and the 95% confidence intervals were shown; the plots and regression models were run separately for each tissue region (AL, BL, AR, BR, C, and D); R software version 3.2.1 was also used for all the analysis.

## Results

**Figure 1:**
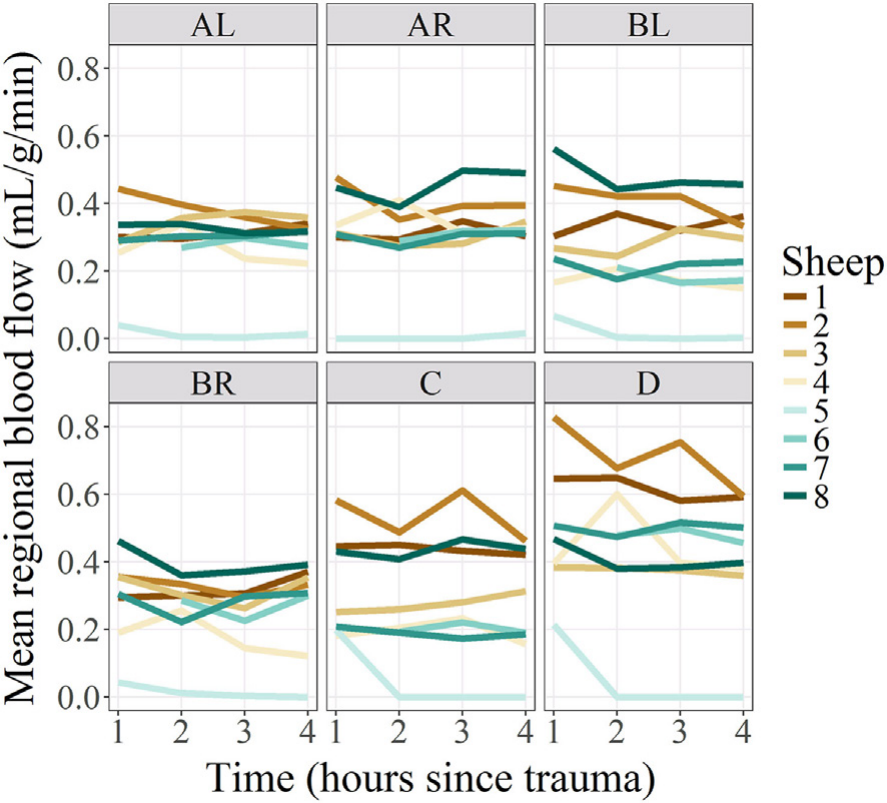
Regional blood flow per regions of interest and subjects, over time. Regional microcirculatory blood flow (RMBF) is represented on the *Y*-axis on a scale of milliliters per gram per minute; time is represented on the *X*-axis at four consecutive time epochs corresponding each of them to 1 h from the injury. Mean RMBF is displayed for each subject with subject number 5 being an outlier as his RMBF was severely reduced.

A convenience sample of eight subjects weighing 40–45 kg was used in this study. Subjects remained cardiovascularly stable throughout the entire study time even after a severe head injury (Table 2), except for one subject (subject number 5) who became profoundly vasoplegic after injury in association with bilateral unreactive midriasis and a comminuted skull fracture. These signs suggested high ICPs leading to herniation state, but due to the significance of his bone fractures, insertion of ICP probes was not feasible, therefore ICP could not be quantified. In addition, this subject showed undetectable RMBF from T1 compatible with a state of global cerebral hypoperfusion (Figure 1).

**Table 2:** Mean arterial blood pressure (mmHg), ICP, and cerebral perfusion pressure (mmHg) values in all subjects at each time point.

MAP/ICP/CPP (mmHg)	T0 preinjury MAP	T1	T2	T3	T4
Subject 1	8,484	80/8/72	81/17/64	86/15/71	99/14/85
Subject 2	100	80/6/72	87/8/79	81/7/73	79/9/70
Subject 3	120	119/7/112	111/7/104	120/8/112	125/9/116
Subject 4	110	124/18/106	122/17/115	120/18/102	119/19/100
Subject 5^a^	73	60/NA	65/NA	69/NA	72/NA
Subject 6	75	90/11/79	96/18/78	88/20/68	114/18/96
Subject 7	70	92/24/68	100/24/76	102/22/80	81/20/61
Subject 8	110	105/40/65	109/32/77	115/34/81	114/35/79
Mean (SD)	1,143 (2,966)	98.6 (17.8)/21.8 (19.2)/82 (19)	101 (14.3)/23.5 (18.6)/84.7 (17.9)	102 (16.9)/24.1 (20)/83.9 (16.7)	104 (18.5)/24.5 (20.9)/86.7 (18.8)

*MAP, mean arterial pressure; ICP, intracranial pressure; CPP, cerebral perfusion pressure, calculated as per MAP–ICP*.

*Only values of MAP are present at T0 as ICP probe was inserted after injury*.

*^a^Subject 5 showed signs of sudden cerebral tamponade with secondary neurogenic shock requiring vasopressor support to maintain MAP. A comminuted skull fracture did not allow for the insertion of PtiO_2_ or ICP probes*.

Systemic variables affecting cerebral perfusion and reflecting peripheral oxygen extraction, such as CO and central venous oxygen saturation (SVCO_2_), respectively, were maintained homogeneously within each subject for the entire study period and with differences among subjects (Table 3).

**Table 3:** Summary statistics for SvO_2_ and CCO data by sheep over all times.

Subject number	*n*	CCO		SvO_2_	
			
		Mean	SD	Mean	SD
SN 01	14,255	6.30	3.62	88.33	5.31
SN 02	15,394	4.17	0.69	77.93	9.65
SN 03	13,222	4.20	0.40	81.65	4.10
SN 04	14,225	4.62	1.20	41.97	11.28
SN 05	14,188	5.38	1.09	80.33	3.30
SN 06	6,061	3.63	0.36	65.94	17.12
SN 08	17,759	3.90	0.92	68.89	5.35
All subjects	95,104	4.76	1.90	81.72	7.31

*Venous saturation of oxygen (SvO_2_) expressed in %*.

*Continuous cardiac output (CCO) expressed in litters per minute*.

Metabolic variables directly affecting oxygen delivery to tissues and cerebral blood volume, such as PaO_2_ and partial pressure of arterial CO_2_, respectively, remained stable throughout the study period for each subject also with minimal variations among subjects (Table 4).

**Table 4:** PH/PCO_2_ and PO_2_ (at FiO_2_ 0.4) values per sheep at each time points.

Subjects	Times				
	
	T0 PH/PCO_2_/PO_2_ (mmHg)	T1 PH/PCO_2_/PO_2_ (mmHg)	T2 PH/PCO_2_/PO_2_ (mmHg)	T3 PH/PCO_2_/PO_2_ (mmHg)	T4 PH/PCO_2_/PO_2_ (mmHg)
Sheep 1	7.40/48/138	7.37/49/138	7.40/49/142	7.40/48/146	7.42/47/136
Sheep 2	7.41/48/141	7.40/49/140	7.43/48/147	7.46/44/152	7.43/41/150
Sheep 3	7.41/48/208	7.40/50/196	7.43/50/193	7.45/48/207	7.45/49/206
Sheep 4	7.49/35/206	7.43/46/220	7.45/45/218	7.49/37/238	7.48/36/239
Sheep 5	7.33/49/128	7.41/44/125	7.35/48/123	7.34/49/103	7.37/48/98
Sheep 6	7.44/38/212	7.43/44/200	7.42/43/222	7.47/40/205	7.44/42/203
Sheep 7	7.35/50/126	7.42/43/171	7.43/47/240	7.49/40/280	7.49/37/266
Sheep 8	7.45/41/203	7.39/49/200	7.42/49/210	7.46/46/217	7.47/44/225
Mean (SD)	7.41 (0.052)/44.6 (5.8)/170 (39.9)	7.41 (0.021)/46.8 (2.8)/174 (35.5)	7.42 (0.030)/47.4 (2.3)/187 (43.6)	7.45 (0.051)/44 (4.5)/194 (56.7)	7.44 (0.039)/43 (4.9)/190 (57.1)

Metabolic variables reflecting oxygen delivery to tissues as well as impacting into microcirculation rheology, such as the hemoglobin level (Table 5) also remained stable at all times in each subject with minor variations among subjects.

**Table 5:** Hemoglobin levels (g/dL) for all subjects over time.

Subjects	Times				
	
	T0	T1	T2	T3	T4
Sheep 1	7.0	7.1	7.2	7.3	7.1
Sheep 2	8.0	8.2	7.9	7.6	8.0
Sheep 3	8.0	7.7	7.7	7.6	7.7
Sheep 4	7.6	8.1	8.0	8.3	8.3
Sheep 5	8.1	9.3	8.2	7.9	7.7
Sheep 6	7.0	7.3	8.1	7.7	7.9
Sheep 7	8.0	7.7	7.8	7.4	7.4
Sheep 8	9.0	8.7	8.8	8.7	8.9
Mean (SD)	7.8 (0.65)	8.0 (0.73)	8.0 (0.46)	7.8 (0.47)	7.9 (0.55)

*Hemoglobin levels were maintained stable from baseline (T0) throughout times (T1–T4) by the completion of a spleen artery ligation*.

Cerebral PtiO_2_ was recorded in every subject from hour 1 after trauma as PtiO_2_ probes were inserted after formalizing craniectomies (Table 6). PtiO_2_ among subjects showed compromised levels of tissue oxygenation through all times except for subject number three which maintained a hyperemic range of values. PtiO_2_ was reflecting only tissue oxygenation at the ipsilateral side to the injury.

**Table 6:** Partial pressure of tissue oxygenation (PtiO_2_) expressed (in mmHg) for all subjects over time.

PTiO_2_ (mmHg)	T1	T2	T3	T4
Sheep 1	1.99	2.69	3.03	3.61
Sheep 2	1.16	11.1	15.21	29.21
Sheep 3	34.01	47.11	48.11	46.23
Sheep 4	16.00	20.21	21.12	24.22
Sheep 5	NA	NA	NA	NA
Sheep 6	11.21	13.31	19.54	22.41
Sheep 7	11.12	10.86	10.31	12.61
Sheep 8	11.01	25.12	26.71	26.41
Median	11.12	13.31	19.54	24.2
IQR	7.1	11.7	11.2	10.3

*T0 represents preinjury phase in which craniectomies are not formalized*.

*PtiO_2_ probes were inserted after trauma, recording PtiO_2_ values only from T1*.

*NA, not available values*.

### RMBF Analysis

Each sheep had a baseline RMBF measure before injury (time 0—T0) and subsequent hourly RMBF measures every hour during 4 h after injury, corresponding to times T1–T4, respectively. RMBF values for each subject and tissue region over the entire study time are represented in Figure 1.

**Figure 2:**
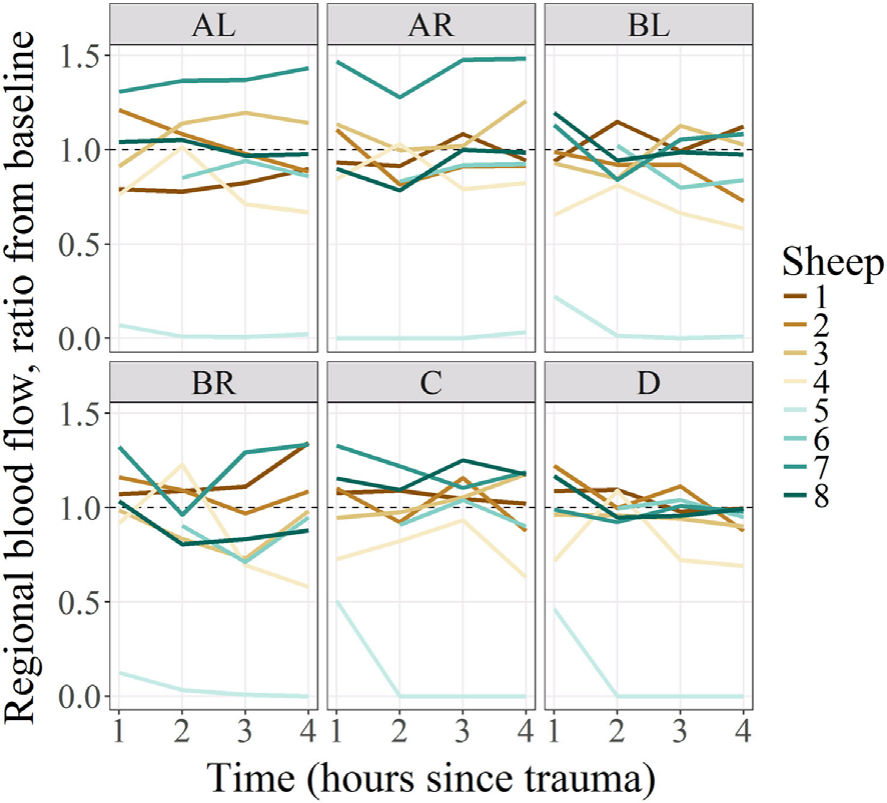
Regional blood flow ratio for all subjects and all regions from baseline. All subjects’ regional microcirculatory blood flow (RMBF) (except for subject 5) was distributed along the ratio of 1. Those above the ratio, indicating that their mean RMBF did increase over time from baseline; those with mean RMBF below the ratio indicating that their mean RMBF was reduced from baseline.

Regional microcirculatory blood flow values for each subject and tissue region over the 4 h from baseline are represented in Figure 2. The ratio of RMBF from 1 to 4 h after injury (T1–T4) compared with baseline (time 0—T0) was also plotted. A ratio of 1 indicated no RMBF changes pre–postinjury. RMBF values below the ratio of 1 represented a reduction in RMBF over time from baseline; RMBF values above such ratio represented an increase in RMBF over time from baseline.

**Figure 3:**
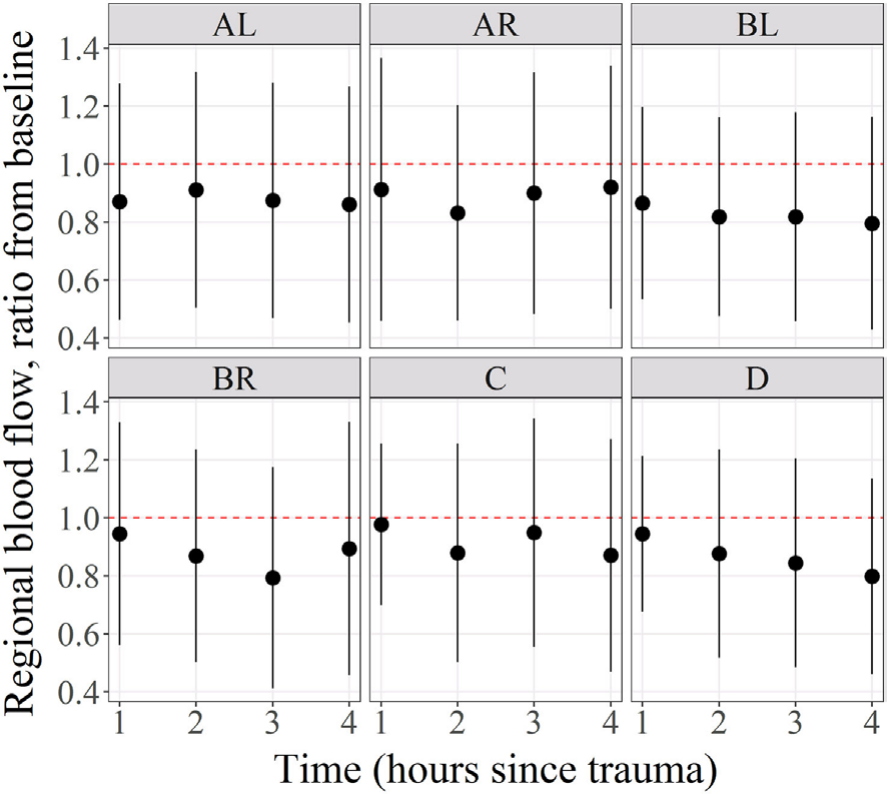
Mean regional blood flow confidence intervals and ratio from baseline. Regional microcirculatory blood flow (RMBF) mean with confidence intervals crossing the ratio of 1 indicated that the changes in RMBF at that region of interest and that time point were not statistically significant.

Regional microcirculatory blood flow means ratio from baseline and for all subjects per anatomical region and time with their 95% confidence intervals represented by the vertical lines were shown in Figure 3. Statistical significance was represented by confidence intervals that would not cross the horizontal reference line of 1. As shown in this figure, the RMBF means at all anatomical regions and times were reduced from baseline but their differences were not statistically significant. Regional flow in the *Y*-axis is represented as per 1 g tissue weight, showing that in our study, physiological RMBF values were found, when normalized to a 100 mg tissue weight.

### APP Scoring

**Figure 4:**
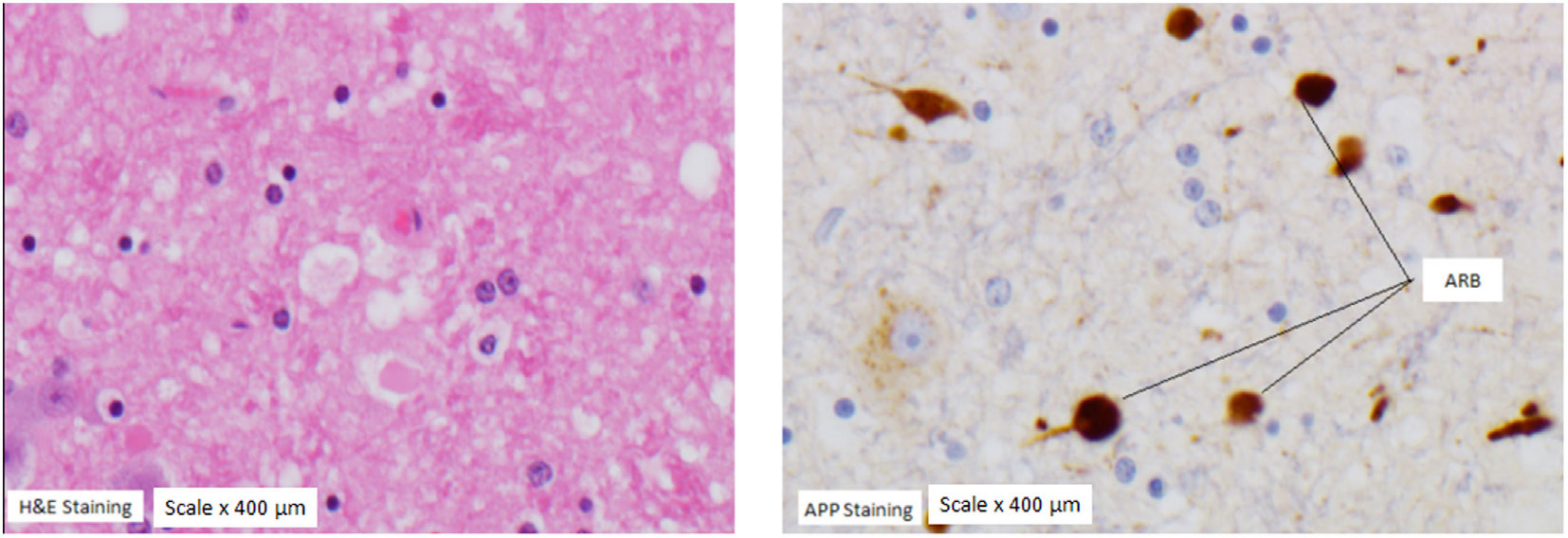
Axonal retraction balls (ARB). ARB under hematoxylin staining (left panel) and under amyloid precursor protein (APP) staining (panel on the right) in a subject with severe APP scoring at the medulla region.

Results for APP scoring are represented in Tables 7 and 8. Minimal APP staining pattern was seen predominantly in all anatomical regions with moderate and severe APP staining pattern also present and distributed homogeneously over all anatomical regions. Evidence of axonal damage and axonal retraction balls (ARB) was found (Figure 4). Wider representation of moderate and severe APP scoring was found among subjects in this study, when compared with a mild head injury cohort.

**Table 7:** Amyloid precursor protein (APP) staining qualitative scores by tissue region in a “severe head injury” model showing its distribution among six of the eight subjects for the severe head injury study.

Anatomical regions	Amount of subjects categorized on each qualitative APP score		
	
	Mild	Moderate	Severe
AL	5	0	1
AR	5	1	0
BL	4	1	1
BR	5	0	1
C	4	1	1
D	5	1	0

*APP staining is predominantly of “mild” category at all regions, but an expression of “moderate” and “severe” is also present among all regions*.

**Table 8:** Amyloid precursor protein (APP) staining qualitative scores by tissue region in a “mild head injury” model showing its distribution among nine subjects for the mild head injury study.

Anatomical regions	Amount of subjects categorized on each qualitative APP score		
	
	Mild	Moderate	Severe
AL	9	0	0
AR	9	0	0
BL	9	0	0
BR	9	0	0
C	2	2	5
D	5	2	2

*APP staining is predominantly “mild” in category at all regions; categories of “moderate” and “severe” are only present at axial anatomical regions where the highest acceleration–deceleration force mainly concentrates*.

### Extracranial Tissues

**Figure 5:**
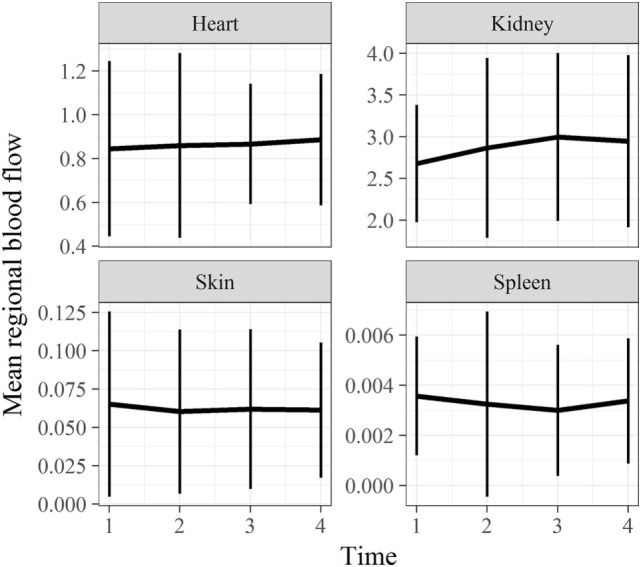
Extracranial tissues’ regional microcirculatory blood flow (RMBF) mean and SD from baseline. Microcirculation at extracranial sites demonstrating an appropriate systemic distribution of spheres. Spleen RMBF was negligible consistent with appropriate splenic arterial ligation.

Direct quantification of RMBF was also performed at extracranial regions, in particular, at the skin, heart, kidney, and spleen (Figure 5). The aim was to demonstrate systemic distribution of color-coded microspheres as a proof of concept during all time points. In addition, RMBF at the spleen aimed to demonstrate that spleen artery ligation had been performed efficiently, demonstrating nearly negligible presence of spheres.

### Statistical Comparison With a Previous Study

**Figure 6:**
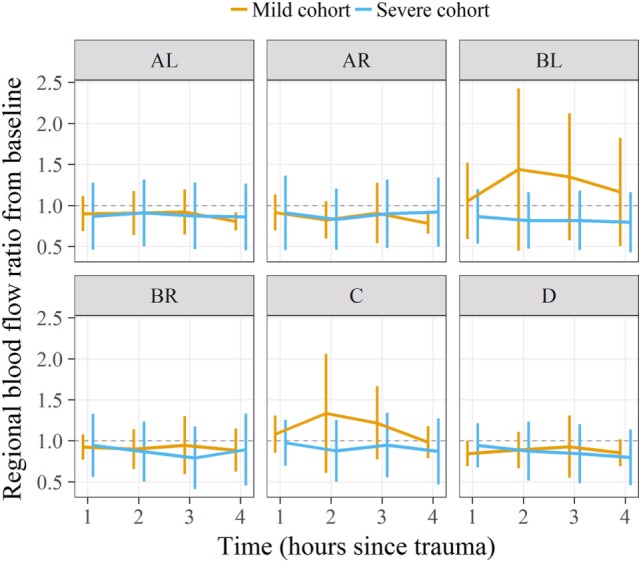
Regional microcirculatory blood flow (RMBF) means and ratio from baseline comparing a “mild” with a “severe” head injury model. On the severe head injury model, RMBF is under the ratio of 1 in all regions of interest and all time points as opposed to the mild head injury where RMBF increases from baseline at the ipsilateral thalamus and pericontusion region.

**Figure 7:**
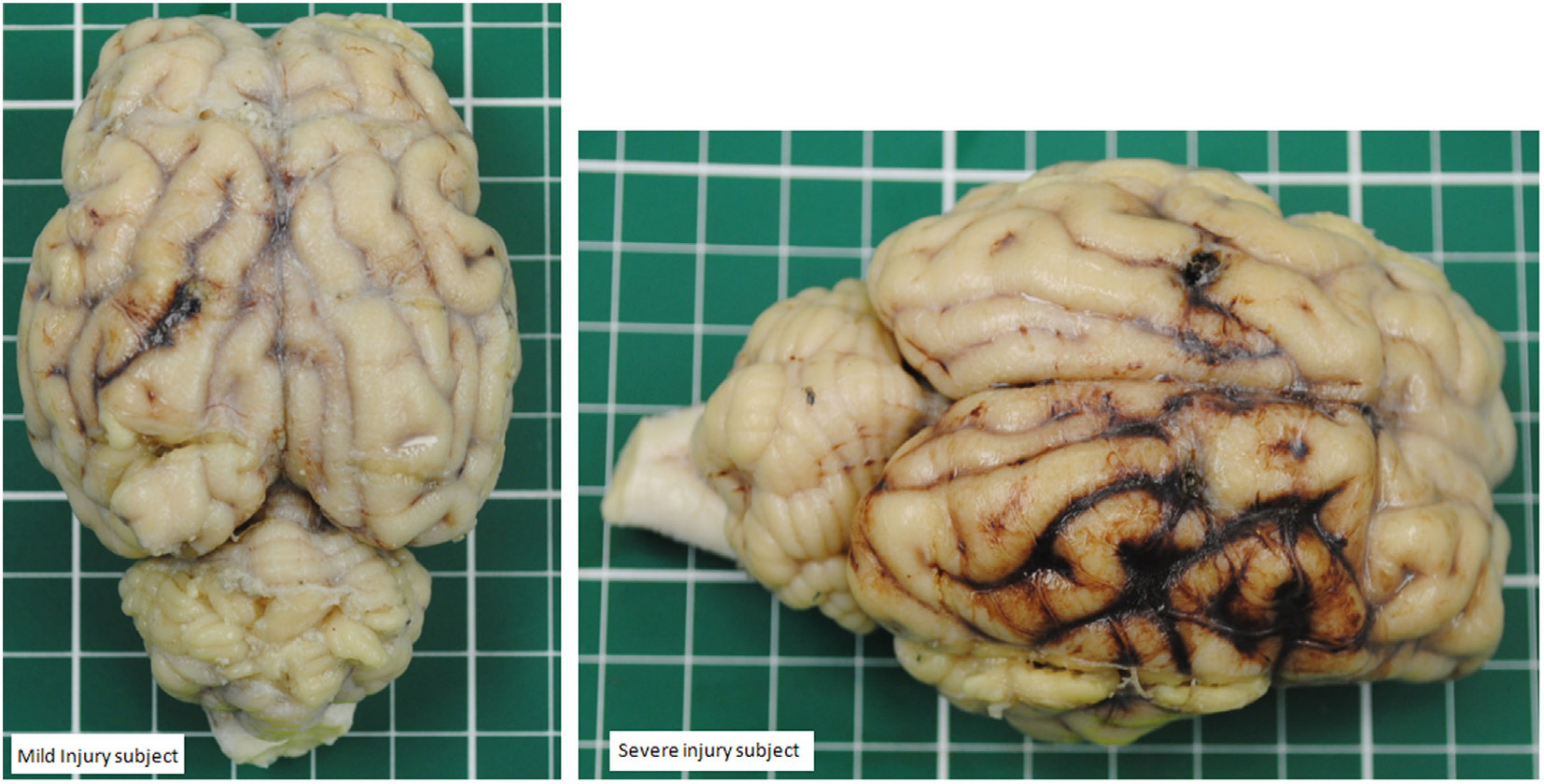
Contrast between a mild head injury contusion (left panel) and a severe contusion (panel on the right) in two different subjects. The mild injury shows a concise contusion at the ipsilateral site of the injury. The severe injury shows a bigger contusion surrounded by hemorrhage and also a contralateral area of hemorrhage.

Statistical comparison of cerebral RMBF at specific anatomical regions of interest and at all times within the “mild head injury” cohort study and the “severe head injury” cohort showed: first, nearly all regions showed a temporal reduction in RMBF from baseline. Second, RMBF at the ischemic penumbra and at the thalamus on the “mild head injury” cohort study had a temporal increase in their RMBF, of uncertain explanation; unlike for the “severe head injury” cohort study in which these areas also showed a consistent reduction of their RMBF over time from baseline (Figure 6) with a contrast of the magnitude of the impact, on two different subjects’ postmortem findings (Figure 7), showing that in the severe injury subject there is an ipsilateral combined with a substantial contra-coup contusion and associated with an evident axial hemorrhage.

The main finding in such comparison was that RMBF in all regions except for medulla, on the “mild head injury” cohort study was consistently higher than on the “severe head injury” cohort study, although these differences were not statistically significant.

Mean ratio of RMBF from baseline by tissue region and study (comparing “severe head injury cohort” with “mild head injury cohort” studies) are shown in Table 9, where none of the differences in ratios between groups were statistically significant as every confidence interval contained the 0 value.

**Table 9:** Mean ratio of regional blood flow from baseline by tissue region and study.

Tissue	Mean	95% confidence interval
AL	0.00	−0.28, 0.28
AR	0.05	−0.25, 0.34
BL	−0.46	−0.99, 0.08
BR	−0.04	−0.32, 0.24
C	−0.22	−0.55, 0.11
D	−0.01	−0.26, 0.23
Heart	−0.32	−0.82, 0.17
Kidney	−0.08	−0.32, 0.16
Skin	−0.66	−2.37, 1.04
Spleen	−0.27	−0.91, 0.37

## Discussion

The main focus of this study is to directly quantify cerebral regional microcirculation in anatomical regions of interest after a severe head injury model which comprises a mixture of contusion and acceleration–deceleration forces, a common mechanism of injury otherwise in real life. In addition, this study contrasts regional flow data with histological data facilitated by the novel experimental model design which isolated adjacent micro-layers of tissue from the same anatomical regions to be separately used for flow and tissue analysis. There are no precedents in this study design. The sequential injection of color-coded microspheres during the experimental phase, allowed a time dependant quantification of cerebral microcirculation at very specific anatomical regions.

This study also captures the hypothetical aim of achieving a “severe” head injury as opposed to a previous study (10) which attempted to recreate a “mild” head injury. Achievement of severe versus mild head injury was intended by using the same impact source (a stunner) while using animals of same gender, age range and species but of smaller weight 40–45 kg on the former versus 65–70 kg weight on the latter and maintaining all other methodological aspects of the study unchanged; those two cohorts of subjects became statistically comparable.

The most important finding in this “severe” head injury study is the reduction of RMBF from baseline (RMBF preinjury or T0) in all subjects, at all anatomical regions of interest and at all times through the study length, as seen in Figures 2 and 3. This finding is consistent with the *a priori* expectations regarding cerebral microcirculation after severe head injury; hence, contrasts with the presence of heterogeneity and temporal variability found in a mild head injury study (10). A consistent reduction in cerebral blood flow is found in all targeted regions during the 4 h of study, although this reduction in RMBF was not statistically significant (Figure 3) likely due to the small sample size; however, it is important to remark that such finding is pathophysiologically and clinically important as it raises multiple issues. Cerebral microcirculation, for example, is consistently jeopardized even in the very early hours after injury despite the preservation of CO, oxygen delivery, and CO_2_. This issue is clinically relevant when considering the potential implications during the resuscitation phase of patients. On the other hand, while statistical significance offers methodological robustness, the experimental nature of this study focuses its relevance in its design and clinical plausibility of findings, both of which are demonstrated.

When cerebral RMBF values were normalized to a global unit of measure (ml/100 g tissue/min), a physiological range of RMBF was found through all anatomical regions.

Furthermore, mean cerebral RMBF at baseline was above the ischemic threshold of 15–20 ml/100 g/min despite having elicited a severe head injury (26). This suggests that in this “severe” head injury model, RMBF although consistently reduced from baseline, was maintained above ischemic thresholds without leading to cerebral infarct. For few of our subjects, this was an unexpected finding, specifically in those cases where the impact had led to compound skull fractures, traumatic midriasis or significant acceleration–deceleration drift of the head. However, one subject did become clinically brain dead and that was supported by the presence of critically low levels of RMBF in few regions and undetected RMBF in other anatomical regions of interest. A possible explanation for the relatively preserved RBMF in this cohort was that despite the applied impact and the fact of having been subjected to a higher degree of injury than in the mild study, this was not accompanied by a worse APP scoring throughout the brain, suggesting that the injury was possible lesser than expected.

Regional microcirculatory blood flow data in this severe head injury study were compared with the study corresponding to a mild head injury (10). No statistical significance was found between the RMBF distribution and quantification throughout all the anatomical regions of interest and during the 4 h of study in either of these two cohorts; however, RMBF in the “severe” head injury study at all regions was lesser than in the “mild” head injury study and the APP staining was more widely distributed, suggesting that our severe injury model could have indeed lead to major degree of injury.

The decline in cerebral RMBF from baseline in this study was not related to an increase in ICP or a reduction in CPP as these parameters were stable and in normal ranges for all subjects except for subjects numbered seven and eight (Table 2). This suggests that reductions in cerebral RMBF postinjury may be also related to early inflammatory changes and tissue disruption after trauma even before the establishment of high ICP. This finding raises concerns related to cerebral vulnerability to ischemia even in the absence of poor intracranial compliance.

This study’s findings and translation into clinical practice are limited by the timeframe of the study, as it focuses on cerebral microcirculation within 4 h after injury. However, as previously emphasized, the novelty of this study design relies on the microcirculatory quantification using cytometric methods when targeting-specific anatomical regions, confirming the reduction of RMBF from the very first hour after trauma. Clinical implications are related to the risk of developing cerebral infarcts even with the co-existence of a preserved ICP, suggesting that efforts should be oriented to optimize cerebral perfusion even within the first hours after head injury, regardless of the level of ICP.

Histopathological analysis using APP staining on the adjacent anatomical regions where flow cytometric count had been performed showed predominantly a minimal degree of APP staining not expected from the severity of the injury applied; however, moderate and severe APP staining scoring was present throughout all regions including the contralateral hemisphere, demonstrating that axonal injury was globally present. In this study, few ARB were found at the medulla region. This is an important finding that supports the higher injury present in this study. ARB are considered to be the hallmark of severe axonal injury. When the axonal cytoskeleton brakes down after injury, axonal transport continues up to the point of such tearing, building up axonal swelling at that disrupted point. When the build-up products alter the cytoskeleton, this retracts back toward the neuronal body leading to a “bulb,” called axonal retraction ball. These findings contrasted with the previous “mild” head injury study (10) in which only medullary and thalamic regions showed a moderate and severe APP staining scoring, confining the axonal injury mainly to axial regions and not to hemispheres (Tables 7 and 8). Axonal disruption can be histologically graded by microscopic quantification of the amount of APP staining and has a good correlation with the intensity of axonal damage (27–30). In this study, even when mild axonal staining predominates over severe; a time-dependent reduction of cerebral RMBF close to ischemic thresholds was found in all regions, from baseline. This is an important finding as it may indicate that even in situations where severe tissue disruption is not derived from the primary injury, there is still the potential for the development of cerebral infarcts. While this theoretical principle is widely accepted in the neurocritical care arena; this study compares and contrasts for first time the interrelation between tissue damage and microcirculation in experimental models at specific anatomical regions, being this the first time that histopathology and quantification of microcirculation are analyzed simultaneously. This study also brings to light the relation between dynamic blood flow changes and the severity of tissue disruption at very specific anatomical regions showing how, flow and tissue, are spatially affected after trauma.

Amyloid precursor protein staining is not a common anatomopathological marker used in the field of neurotrauma, but it is reproducible and its applicability has been validated. It is used to quantify and define the distribution of axonal damage, as demonstrated in this study.

Cytometric analysis in other organs, in particular the skin and spleen is shown in Figure 6. RMBF in spleen showed negligible perfusion as expected from the arterial spleen ligation technique completed in the preinjury phase. Spleen ligation allowed the maintenance of a steady-state level of hemoglobin, particularly during a stress response phase when splenic red blood cell storage is well described in ovine models (17). Splenic RMBF was expressed in milliliters per gram per minute, varying between 0.0030 and 0.0036 ml/g/min, a thousand times less than normal controls (12). This finding confirms that our splenic artery ligation was successful as well proven by the necrotic aspect of the spleen after harvesting. The main effect derived from the arterial spleen ligation is found on the stability of hemoglobin in all subjects and over all times after injury (Table 5); suggesting that the temporal changes on cerebral RMBF and PtiO_2_ seen in our subjects were not related to anemia. This is an important finding as this reflects the state of cerebral microcirculation after trauma in conditions of baseline hemoglobin, a significant confounder in microcirculation. But, while clinical extrapolations are not possible considering the experimental nature of this study, it is important to highlight that the hemoglobin levels in this cohort of subjects are to a degree higher to those currently accepted within clinical practice, especially among physicians tolerating a restrictive transfusion threshold (4). This raises the concern of the potential risk of cerebral ischemia after trauma if anemia was present.

Cerebral oximetry data were provided by PtiO_2_ in all subjects. These probe sensors were introduced *via* a burr hole completed immediately after injury, with the intention not to bias the intracranial compliance, particularly if the opening of the dura had been performed before injury. In this study, PtiO_2_ values in four of the eight studied subjects (SN 3, SN 4, SN 6, and SN 8), maintained levels of tissue oxygenation above 15 mmHg, suggesting that oxygen delivery to the brain was preserved despite the reduction of RMBF from baseline. One subject (subject 5) had no recordable PtiO_2_ as probes could not be safely introduced due to the presence of compound cranial fractures. These findings may indicate that despite the global reduction in RMBF from baseline, the maintenance of above-ischemic perfusion thresholds may be sufficient to preserve tissue oxygenation, emphasizing the relevance of preserving cerebral perfusion even from early hours post trauma. Of significant relevance is to note that RMBF does not apply to the state of CPP but to the preservation of cerebral microcirculation, as demonstrated in this study.

### Study Limitations

The biggest limitation of this study relies on the limited capacity to clinically extrapolate conclusions raised from experimental models, in general. However, it is from experimental studies that direct quantification of cerebral microcirculation, as described in this study, becomes feasible. Another important limitation relates to the restricted length of time during which the animals were monitored and studied (4 h postinjury). Hence, although longer monitoring time could have demonstrated a wider range of pathophysiological processes affecting microcirculation; the focus of this study was to capture the early changes in RMBF at specific regions of interest contrasted to the early histopathology changes derived from axonal injury, directly related to trauma.

An additional limitation relates to the small sample size leading to an under-power study. However, it is worth considering that for this longitudinal study design, five observations per sheep (consistent to the five time points during which data were captured) were performed to maximize the statistical power from the relatively small number of subjects. Therefore, the authors believe that the methodological aspect of this study is robust and valid. Moreover, the aim of this experimental study lays on the fact of assessing a pathological process in a manner that is not clinically possible or feasible; therefore its findings become of clinical relevance and contribute to the pool of current knowledge.

An additional limitation relies on the concept of “severe” head injury and the comparison with a previous study, considered to be representing a “mild” injury. The authors acknowledge that the assumption of “severe” stands upon a theoretical rationale as the method was a reproduction from the previous study with the only variation being the weight of the subjects. So, while the reduction on the quantification of flow is statistically not significant, when combined with the macroscopic and histologic findings the authors can only suggest that these subjects had been subjected to a bigger impact; however, this does not imply being a “severe” head injury as we could not detect sufficient signs to support it.

Finally, the APP staining score was not statistically compared or correlated to the RMBF quantification because it would have been of an arbitrary value. While APP staining is a validated tool used in clinical settings, RMBF quantification is mainly used at experimental scenarios and therefore the authors found no substantial reason to statistically compare both parameters, as the value of that would be of dubious value. Instead, simply contrasting axonal integrity with cytometric quantification of cerebral microcirculation during the acute phase of head injury, offers a theoretical perspective of an otherwise, not approachable method.

## Conclusion

After severe head injury, cerebral microcirculation at the ipsilateral and contralateral site of a contusion in addition to the ipsilateral thalamus and medulla shows a consistent decline over the first 4 h after injury, when compared with baseline. The widespread reduction on cerebral microcirculation occurs independently of cerebral perfusion pressure and ICP and is present despite cardiovascular stability. Although not statistically significant, a temporal reduction on cerebral microcirculation contrasts with the relatively spared axonal integrity represented by the APP staining; this may suggest that even in relatively severe head injury, microcirculation is globally reduced from baseline.

## Ethics Statement

Approval for this study was obtained from the Animal Ethics Committee of the Queensland University of Technology and the University of Queensland, Australia.

## Author Contributions

Primary roles: JB led the study design, surgical procedures, data collection, data interpretation, and manuscript preparation. KC undertook the histopathology analysis. KD led all laboratory support and contributed to the manuscript preparation. SD orchestrated all surgical procedures and data collection. DP and OR performed the intracardiac echography and transeptal catheterization. LG overviewed the manuscript preparation. AB performed the statistical analysis. JP, RB, and JF reviewed the interpretation and manuscript preparation.

## Conflict of Interest Statement

The authors declare that the research was conducted in the absence of any commercial or financial relationships that could be construed as a potential conflict of interest.
